# Case Report: Neonatal nephropathy with polycystic appearance in child harboring WT1 variant

**DOI:** 10.3389/fped.2025.1693036

**Published:** 2025-12-10

**Authors:** Tuyen Thi Thanh Nguyen, Quynh Thuy Huong Tran, Huong Thi Thanh Nguyen, Trang Quynh Mai Pham, Doan Tran, Kenji Fukui, Hiroyasu Tsukaguchi

**Affiliations:** 1 Neonatalogy 2 – Metabolism – Genetics Department, Children’s Hospital 1, Ho Chi Minh City, Vietnam; 2 Department of Genome Editing, Institute of Biomedical Science, Kansai Medical University, Hirakata, Japan; 3 Division of Nephrology, Second Department of Internal Medicine, Kansai Medical University, Hirakata, Japan; 4 Department of Food Science and Nutrition, Faculty of Human Life and Environment, Nara Women’s University, Nara, Japan; 5 Clinical Genetics Center, Kansai Medical University Hospital, Hirakata, Japan

**Keywords:** Wilms tumor, renal failure, cystic kidney, gonadal development, collagen

## Abstract

**Background:**

Mutations of the Wilms tumor suppressor-1 gene (*WT1*) cause a WT1-related nephropathy characterized by a triad of glomerulopathy, defective genital development, and Wilms or gonadal tumor. *WT1* mutations affecting the second to third zinc finger motifs (residue 428–511) often lead to infantile-onset glomerulopathy that progresses rapidly to end-stage renal failure by the age of 2.5 years. Most of these cases have been reported as Denys–Drash syndrome (DDS). We report a patient harboring a WT1 p.Arg467Gln variant who developed a severe neonatal-onset renal failure since birth, with an unusual cystic pattern of kidney enlargement on ultrasound.

**Case presentation:**

A 22-day-old neonate manifested acute anuric renal failure shortly after birth. Renal ultrasound revealed moderately enlarged, bilaterally hyperechoic kidneys (+2.0 to +3.0 SD), the appearance of which resembled that of polycystic kidney disease. The patient showed normal male external genitalia development except for undescended testes. There were no abnormalities in the hepatobiliary duct systems or lungs. The occurrence of the same cystic kidney disorder in the elder sibling born to healthy parents suggests germline mosaicism. He died from multiorgan failure on postnatal day 47.

**Genetic results:**

The next-generation sequencing (NGS) screening panel analysis of 4,503 known disease genes revealed a heterozygous pathogenic WT1 p.Arg467Gln variant, which has been reported elsewhere in children formerly categorized under the DDS subtype. The Arg467 residue is the most frequent site of mutations in DDS and is predicted to hinder the binding of the third zinc finger to DNA. Additionally, a heterozygous *COL4A4* p.Gly864Val missense variant of uncertain significance (VUS) was detected. Genome-wide copy number analysis did not detect any structural abnormalities.

**Conclusion:**

Our observation highlights two aspects of the WT1 p.Arg467Gln variant in WT1-related nephropathy: (1) The p.Arg467Gln variant causes severe neonatal-onset nephropathy likely through a potent dominant-negative inhibition, and (2) it may manifest a cystic/dysplastic renal phenotype in the presence of coexisting cytogenic modifiers. Future studies are necessary to assess the relevance of the *COL4A4* variant in cystic disease and to explore hidden modifier genes through deep sequencing.

## 1 Introduction

Over the past several decades, patients with mutations of the Wilms tumor suppressor-1 gene (*WT1*) have been categorized into three major subtypes: Denys–Drash syndrome (DDS), Frasier syndrome, and WAGR (Wilms tumors, aniridia, genitourinary anomalies, retardation) syndrome, primarily based on the phenotype (1–3). DDS is characterized by a triad of glomerulopathy, abnormal gonadal development, and malignancies (Wilms tumor and gonadoblastoma) (4, 5). Patients previously diagnosed with DDS exhibit glomerulopathy (95%), which typically manifests as steroid-resistant nephrotic syndrome (SRNS) with focal segmental glomerulosclerosis (FSGS) or diffuse mesangial sclerosis (DMS) histology. The average age of onset is 1.4 years, and the disease progresses to end-stage renal disease (ESRD) before the age of 4 years (6–8). Wilms tumor is found in ∼38%–60% of DDS patients and generally presents at an average age of 1.3–1.6 years (4, 7).

Recent genetic studies revealed that DDS subtypes are mostly caused by missense variants affecting the second and third zinc finger DNA-binding domains. The DDS-causing variants cluster into the hotspot residue arginine 467, which impairs *WT1*–DNA interactions. The phenotypes of WT1 patients are remarkably diverse concerning both severity and morphologic pattern. Recent advances in clinical genetics prompted a genotype-first diagnosis of WT1 disorders. WT1 disorders leading to a spectrum of phenotypes are now integrated into a single entity of “WT1-related nephropathy” (4).

Autosomal dominant polycystic kidney disease (ADPKD) is the most common inherited kidney disorder, typically presenting in adulthood and most often caused by *PKD1* or *PKD2* mutations. In contrast, cystic kidney disease in neonates is rare and more characteristically associated with autosomal recessive PKD (ARPKD, PKHD1-related). However, early-onset or atypical cystic phenotypes may also arise from additional pathogenic variants or modifier effects in other developmental genes, including *WT1*.

We report a 22-day-old male neonate, harboring a WT1 p.Arg467Gln variant located in exon 9, encoding the third zinc finger motif, the amino acid position of which has been previously linked to the clinical subtype of DDS. The patient presented with bilateral enlarged and echogenic kidneys, resembling an ultrasonographic pattern of polycystic kidney disease (PKD). He manifested ESRD at birth and mild gonadal maldevelopment (ectopic testes). Targeted exon-panel sequencing revealed no mutations in *PKD1*, *PKD2*, and *PKHD1*, suggesting the contribution of these genes to cyst formation is less likely. Notably, the sequencing revealed a heterozygous *COL4A4* variant of uncertain significance (VUS) in the context of Alport syndrome (Table 1). Our case suggests that the cystic change may manifest as a subtype of WT1-related nephropathy, particularly when the second modifier, such as *COL4A4* variant, coexists.

**Table 1:** Genetic variants detected in the proband (II-3).

Gene	Variants	
	*WT1*	*COL4A4*
Cytogenetic position	11p13	2q36.3
Position (GRCh38.p14)	32392019	227056070
Reference cDNA sequence	NM_024426.6	MN_000092.5
	c.1400G>A	c.2591G>T
Amino acid change	p.Arg467Gln^a^	p.Gly864Val^b^
dbSNP ID	rs121907903	NA
Allele frequency	ND^c^	ND
Zygosity	Heterozygote	Heterozygote
ClinVar	VCV000419332.19	NA
ACMG classification	Pathogenic	VUS

ND, not detected; NA, not appreciated.

a The nomenclature of p.Arg467Gln is according to the Reference NM_024426.6 (isoform D). The p.Arg467 substitutions have been originally reported as alternate variant designation p.Arg394 (32).

b Missense variant that substitutes the same Gly codon 864 from our case into a distinct amino acid (glycine to arginine, c.2590G>A, p.Gly864Arg) is reported in ClinVar VCV000550588.8 (pathogenic) and dbSNP (rs937550597).

c The variants are absent in large population cohorts (gnomAD, Asian).

## 2 Case presentation

**Figure 1:**
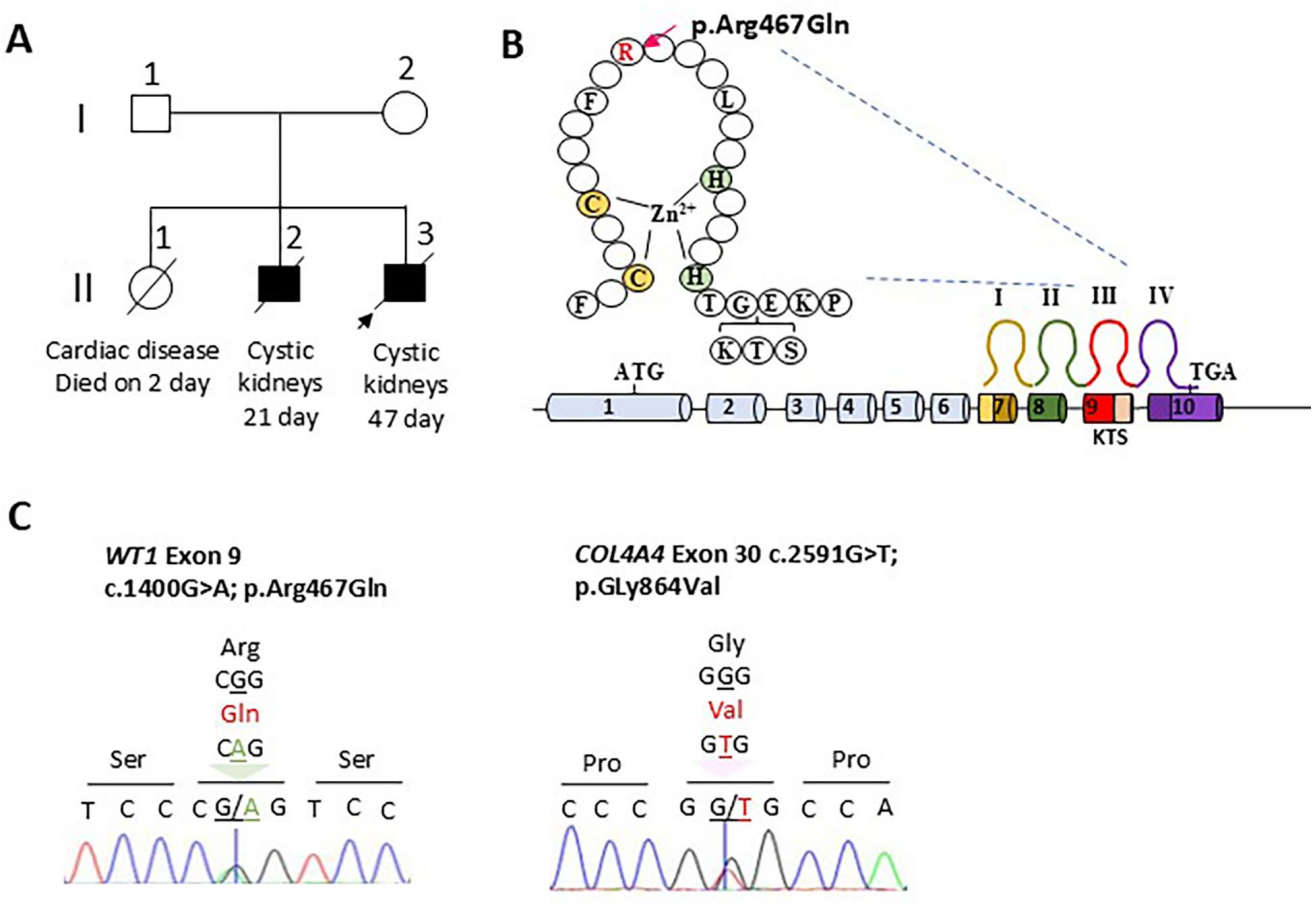
Clinical and genetic features of the patient with WT1 p.Arg467G1n. **(A)** Pedigree. The proband (arrow, II-3) presented with bilaterally enlarged kidneys and deceased on postnatal day 47. The father (I-1) and mother (I-2) are healthy. The second child (II-2) exhibited neonatal nephropathy with a cystic kidney appearance similar to the proband (II-3) and died on day 21. The first child (II-1) died from cardiac complications on day 2 and lacked any apparent renal abnormalities. **(B)** Domain structures: the human WT1 protein comprises four C_2_H_2_ zinc fingers (I–IV; C, cysteine; H, histidine). Exons 7–10 encode zinc fingers I–IV, with the three amino acids lysine, threonine, and serine (KTS) splice site located at the 3′ end of exon 9. The p.Arg467GIn variant lies within the third zinc finger. **(C)** Sanger validation: Sanger sequencing of the proband confirmed two heterozygous missense variants: WT1 p.Arg467GIn (NM_024426.6, dbSNP121907903) and COL4A4 p.Gly864Val (NM_000092.5).

The proband was a 22-day-old male neonate (II-3), the third child of a healthy non-consanguineous marriage (age: father, 40; mother, 39) (Figure 1A). The first child, a girl, died on postnatal day 2 due to congenital heart disease. The second child (II-2), a phenotypic male, died from severe neonatal nephropathy with a polycystic ultrasound appearance similar to the proband (II-3) at the age of 21 days. He was born at full term, 39 weeks of gestation, via spontaneous vaginal delivery after an uneventful pregnancy. Apgar scores at 1 and 5 min were 7 and 8, respectively. Birth weight was 2.5 kg. Amniotic fluid was normal in appearance and amount during the pregnancy.

At 22 days of life, he was admitted due to anuria and generalized edema. For the 10 days before admission, vomiting and diarrhea persisted. On admission, he weighed 3.2 kg. Physical examination revealed abdominal distension and pitting edema in the extremities. The external genitalia were male-type, while the testes were not palpable in the scrotum. A 6 Fr transurethral catheter was inserted and drained ∼5 mL of urine, confirming urethral patency without hypospadias. No jaundice, hepatosplenomegaly, facial dysmorphism, or limb malformation was noted. Since the first day of hospitalization, he developed respiratory distress and progressed to respiratory failure requiring mechanical ventilation (Supplementary Figure S1). No distinctive facial characteristics or limb deformity suggestive of Potter sequence was seen.

**Figure 2:**
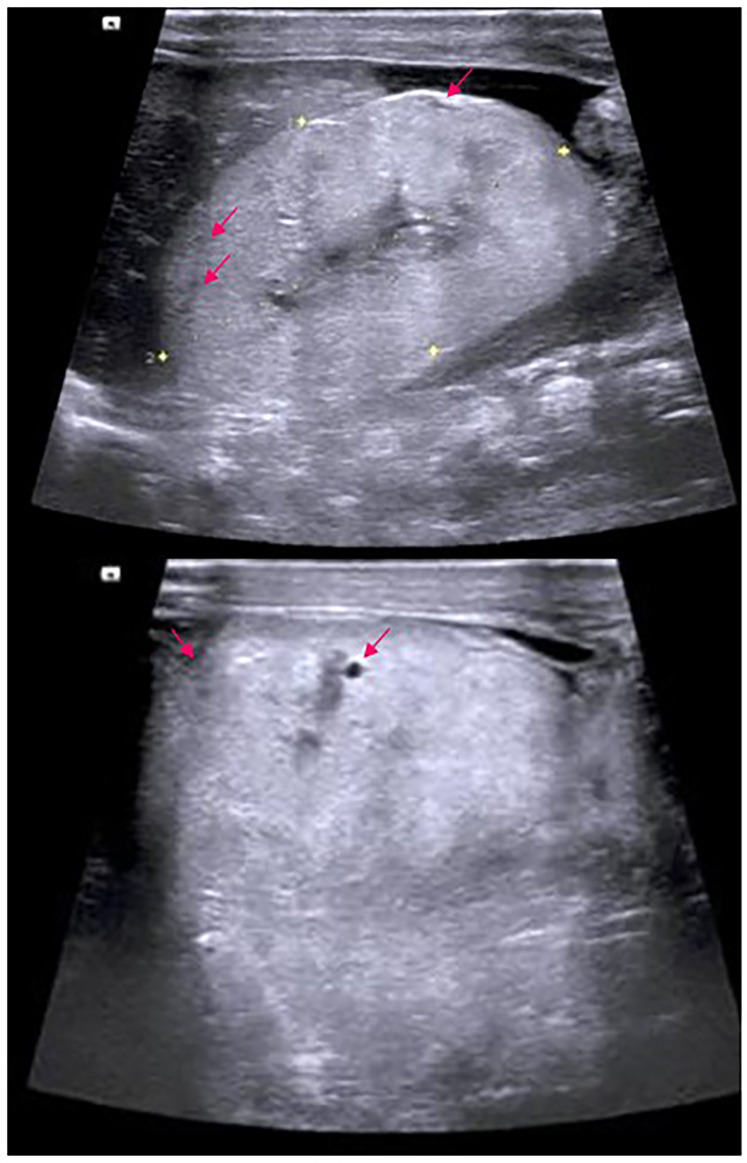
Renal ultrasonography in a child with WT1 p.Arg467GIn. Ultrasonography of the proband (II-3) at 22 days of age shows bilaterally enlarged kidneys with hyperechoic, thickened parenchyma (right kidney: 54 mm × 32 mm, top panel; left kidney: 53 mm × 36 mm, lower panel). The kidneys retain a grossly normal overall shape but showed poor corticomedullary differentiation. Multiple cysts of varying sizes (arrows) are scattered throughout the renal parenchyma.

Laboratory tests revealed anemia (Hb 9.4 g/dL), hyperkalemia (6.4 mmol/L), hypoalbuminemia (1.9 g/dL), hyponatremia (105.8 mmol/L), hypocalcemia (0.8 mmol/L), and severe renal dysfunction (serum creatinine 734.3 µmol/L). Liver functions, including bilirubin levels, were within normal ranges. Arterial blood gas showed metabolic acidosis with respiratory compensation (Supplementary Table 1). Ultrasound revealed a bilateral kidney enlargement (right kidney, 32 mm × 54 mm; left kidney, 36 mm × 53 mm), which ranged within +2.0 to +3.0 SD compared with age-matched controls (9–11). Both kidneys showed parenchymal hyperechogenicity with poor corticomedullary differentiation and scattered microcysts of variable size (approximately 2.5 mm in diameter) (Figure 2). Ultrasonography further revealed that the liver is normal in size, shape, and parenchymal echotexture (including portal vein and bile ducts). No hepatosplenomegaly, fibrosis, or cystic lesions were found. The testes were absent in the scrotum and retained in the peritoneal cavity, confirming the diagnosis of undescended testes (Supplementary Figure S2).

Despite intensive treatment including antibiotics, peritoneal dialysis, and blood transfusion, renal function worsened, leading to respiratory distress and death at 47 days of age (25 days post-admission) (Supplementary Figure S1).

### 2.1 Genetic analysis for the proband (II-3)

Genome-wide copy number analysis revealed no structural abnormalities, consistent with a 46, XY chromosome arrangement (Supplementary Figure S3).

NGS analysis of the proband (II-3) using the G4500 panel, which targets 4,503 clinically relevant genes (12), identified two heterozygous missense variants (Table 1). The first variant is a *WT1* missense mutation: NM_024426.6; exon 9; c.1400G>A (p.Arg467Gln), affecting a known hotspot residue within the third zinc finger domain and previously associated with severe WT1-related nephropathy (formerly classified as DDS subtype) (Figure 1B) (2, 13). According to the American College of Medical Genetics and Genomics (ACMG) guidelines, p.Arg467Gln is classified as pathogenic (Table 1). Three-dimensional structural modeling suggests that p.Arg467Gln destabilizes the interaction between the third zinc finger and target DNA, which potentially impairs WT1 transactivation (Supplementary Figure S4).

The second variant is a heterozygous *COL4A4* missense substitution: NM_000092.5; exon 30; c.2591G>T (p.Gly864Val). Although this variant has not been reported in ClinVar, a different substitution at the same residue (p.Gly864Arg) is classified as VUS according to the ACMG criteria (Table 1, Supplementary Table S2). The p.Gly864Val variant is absent from gnomAD. Sanger sequencing confirmed both *WT1* and *COL4A4* variants in the proband (Figure 1C). No pathogenic variants were detected in *PKD1*, *PKD2*, or *PKHD1*.

## 3 Discussion

### 3.1 Renal ultrasonographic findings and review of literature

We report a neonate with WT1-related nephropathy presenting with bilaterally enlarged echogenic kidneys resembling the pattern of polycystic kidney, without hepatic abnormalities or extrarenal cysts. These findings suggest that cystic appearance nephropathy is associated with WT1 dysfunction, rather than typical PKD.

The patient's kidneys were moderately enlarged (+2 to +3 SD) with poor corticomedullary distinction (9). Such features may represent cystic changes in WT1-related nephropathy or concurrent primary cilia disorders caused by mutations in cilia proteins. In contrast to ARPKD, which usually exhibits massive kidney enlargement (+4 to +6 SD) and liver fibrosis, early-onset autosomal dominant PKD (ADPKD) typically presents with mild enlargement (+1 to +2 SD) and preserved corticomedullary differentiation (Supplementary Table S3).

Only a few reports have described renal ultrasound findings in patients with the WT1 p.Arg467Gln variant. The kidneys of our patient are larger than the other two cases. First, Yoshino et al. described much smaller kidneys (ranging from −1.0 to −4.0 SD compared with age-matched controls) with increased parenchymal echogenicity in the absence of cysts, suggesting the presence of dysplastic changes (14). Second, in Maalouf et al.'s study, MRI revealed that the degree of enlarged kidneys was close to that of our case, while lacking any visible cysts in the parenchyma (Supplementary Table S4) (15).

The unusual cystic kidney appearance in our patients highlights the need to explore an underlying second modifier as a potential mechanism linking WT1 variants and cyst formation.

### 3.2 Mechanisms of cystic kidney appearance in a child with the third zinc finger of the *WT1* variant

#### 3.2.1 Role of the *WT1* variant affecting the zinc finger motifs

*WT1* mutations located within the second to third zinc finger domains cause severe WT1-related nephropathy, clinically classified as a DDS subtype. In terms of nosology, the WT-1 disorders have been integrated into an entity known as “WT1-related nephropathy” based on the molecular diagnosis. The clinical “DDS” subtype, which represents a subtype arising from mutations within the second to third zinc finger, may still be used in some literature. Clinically, DDS is characterized by a triad of early-onset glomerulopathy, typically manifesting as SRNS progressing to ESRD before the age of 4 years, gonadal development defect, and Wilms tumor predisposition. In our patient, the gonadal defect was relatively mild, showing undescended testes only. However, because of the earlier death, we could not have examined the histology of the developing nephrons, as well as Wilms tumors.

The unique feature of our case is bilateral kidney enlargement with multiple microcysts, representing a polycystic appearance that is unusual for WT1-related nephropathy. The mechanism by which *WT1* variants result in a PKD-like pattern remains unclear. The co-occurrence of the *PKD1* variant with the *WT1* variant has been reported in several cases. For example, bilateral PKD-like kidneys were reported in a child with *PKD1* hypomorphic mutations (p.Lys4147Glu) along with a WT1 zinc finger variant (p.Asp469Asn) (16). The patient developed a unilateral cystic Wilms tumor at the age of 22 months. Two WAGR cases, who had 11p13 deletion encompassing *WT1*, showed PKD-like kidneys (17, 18). Notably, in a rare PKD family (*PKD1*, p.Glu2771Lys), the cystogenesis was attenuated in the presence of a concurrent WT1 Frasier subtype variant (c.1432+4C>T), suggesting the modifier effects may vary depending on the combination of digenic alleles (19). The data collectively suggest that the WT1 zinc finger variant could show the cystic kidney appearance when other cystogenic modifiers coexist.

Our patient carried a missense variant (p.Arg467Gln) in the third zinc finger, which represents a mutation hotspot and a crucial site for DNA binding and transactivation of key genes involved in renal and gonadal development (Supplementary Figure S5). The p.Arg467 residue lies within the zinc finger 3 domain, which is a mutation hotspot and is critically involved in DNA binding and transcriptional regulation (20). The most frequently reported substitution, p.Arg467Trp, is typically associated with the classical DDS phenotype that progresses to ESRD in early childhood. In contrast, the rare p.Arg467Gln variant appears to confer even more severe renal phenotype with earlier onset. The observation supports the notion that different amino acid substitutions at the identical 467 residue may have a distinctive structural effect on WT1 protein conformation and DNA-binding affinity. Together with our findings, these data support emerging evidence that p.Arg467Gln may represent one of the most deleterious mutations within the zinc finger region of WT1.

In contrast, the truncating mutations located in the second zinc finger domains (exon 8, p.Arg435Ter, previously reported as p.Arg362Ter), which introduce a stop codon and delete the last three zinc finger domains, cause the Wilms tumor at age of 1 year in all 11 reported cases, but 8 of these patients lacked any nephropathy manifestations as of the publication date (14). Missense mutations in the zinc finger motifs produce notably more severe nephropathy with faster progression to ESRD at the age of 0.9 years, while manifesting the Wilms tumors in 50% of all the cases at the age of 1.3–1.6 years (21). These observations suggest that the third zinc finger missense variants in our case exert a potent dominant-negative effect on the DNA binding, thereby disrupting normal nephron development more severely than mere haploinsufficiency. Although no cystic appearance has previously been reported in children with WT1 p.Arg467Gln, our findings raise the possibility that the *COL4A4* variant may contribute to a cystic phenotype in WT1 nephropathy. Further study on the mechanisms linking *WT1* variants and renal cyst formation is warranted.

#### 3.2.2 Role of the *COL4A4* variant

Notably, our case with p.Arg467Gln WT1 variant had an additional missense COL4A4 p.Gly864Val variant. Type IV collagen alpha consists of six subunits (α1–α6), producing a heterotrimer network (1) α1–α1–α2, (2) α3–α4–α5, and (3) α5–α5–α6 (22). The isoform switch of type IV collagen, from fetal form α1–α1–α2 to adult form α3–α4–α5, occurs during embryonal nephrogenesis. The α4(IV) subunit is present at 11 weeks in human embryonal kidneys and is a major component of the glomerular basement membrane (GBM) of mature glomeruli, with some minor expression along the distal tubules (22–24).

The role of the heterozygous *COL4A4* VUS variant in the cystic formation of WT1-related nephropathy kidney remains unclear. However, several lines of evidence support the possible involvement of the *COL4A4* VUS variant in the cystic kidney phenotype. First, a recent genetic study with ADPKD and Alport syndrome revealed that *COL4A4* variants may confer susceptibility to cystic changes (25). Second, *COL4A4* is focally expressed in the tubular BM to a lesser degree than in the GBM. The *COL4A4* variant may affect the stability and strength of the tubular cell–matrix interaction, rendering the tubular cells more susceptible to cyst formation. Third, in human organoids with *WT1* variants, expression of *COL4A3* and *COL4A4* is downregulated (26–28).

Since the kidney cysts in Alport syndrome and the no-mutation-detected subtype of ADPKD are usually smaller and fewer than typical ADPKD, the role of *COL4A3* and *COL4A4* in tubular basement membrane might be only minor in the cystogenesis. WT1 does not directly bind to the 5′ regulatory region of the *COL4A3* and *COL4A4* genes. However, WT1 acts as a “master regulator” of nephron development by coordinating the collagen subunit assembly with other broader programs controlling epithelial differentiation, cell adhesion, and extracellular matrix interaction.

Collectively, the COL4A4 p.Gly864Val variant may potentially act as a gene modifier predisposing to the cystic renal phenotype in the presence of another pathogenic variant like WT1. Further study is necessary to verify the role of the *COL4A4* variant in cystogenesis.

### 3.3 Genetic interaction and di- or oligogenic model

A second genetic modifier may accelerate the cystic changes. This hypothesis is supported by the reports of rare cases of *WT1* mutation with cystic kidney pattern in the coexistence of *WT1* mutation and a cystogenic gene (19). Such observations raise the possibility that the hidden, cystogenic gene variants contribute to the severe renal phenotypes, which might have been overlooked in our NGS panel (Supplementary Figure S6) (29, 30). Previous studies showed that severe prenatal PKD patients may carry, in addition to the inherited PKD mutation, a co-inheritance of ADPKD and ARPKD (oligogenic model of PKD) or other cystogenic genes such as *HNF1B* (15) or a ciliopathic gene such as *IFT140* (31). In the present case, the recurrence of severe cystic renal phenotypes in two siblings (II-2 and II-3) born to unaffected parents suggests a shared monogenic etiology. The likelihood of identical *de novo* mutations arising independently in two consecutive pregnancies is extremely low (incidence of 10^−12^–10^−14^). Therefore, an alternative explanation could be germline mosaicism in either parent, as previously reported in familial cases of cystic kidney disease (4).

### 3.4 Limitation

The limitation of this study is that the patient died at the age of 1.5 months before reaching the average onset age of Wilms tumor in patients with WT1-related nephropathy of DDS subtype. Therefore, we could not assess the renal histology to determine whether the child developed the tumor or dysplastic changes. Consequently, our observations rely solely on the ultrasonographic phenotype, which displayed an unusual cystic kidney appearance. While the imaging modalities, such as MRI or CT, and kidney biopsy can aid differential diagnosis, they are often not feasible in acutely ill neonates, prioritizing the ultrasonography examination for clinical assessment.

Finally, although our panel sequencing targeted most previously known cystogenic genes, it did not cover all relevant genes (Supplementary Figure S6). Therefore, additional modifiers may have been missed. Our case underscores the importance of further genetic testing, such as whole-exome or whole-genome sequencing, for not only accurate diagnosis but also genetic counseling for affected families.

## 4 Conclusion

We report a neonate with the WT1 p.Arg467Gln variant who presented with markedly enlarged, cystic-appearing kidneys. The p.Arg467Gln variant appears to have potent dominant-negative effects and may increase the susceptibility of kidney developmental defects resembling PKD when additional modifiers (e.g., the *COL4A4* variant) coexist (Supplementary Figure S7). Future *in vitro* and *in vivo* studies are needed to clarify the mechanisms of this variant in renal development.

## Data Availability

The data supporting the findings of this case report are derived from clinical records of the patient and cannot be made publicly available due to privacy and confidentiality restrictions. All relevant information has been included within the article. Additional de-identified details may be provided by the corresponding author upon reasonable request and with approval from the involved institutions.
