# Supplementary material for: Case Report: Neonatal nephropathy with polycystic appearance in child harboring WT1 variant
Source: Front Pediatr. 2025 Dec 10;13:1693036. doi: 10.3389/fped.2025.1693036 (PMC12728025; doi:10.3389/fped.2025.1693036)
Supplement: Supplementary file 1 [file Presentation1.pdf]

Supplementary Figure and Table

Neonatal Nephropathy with Polycystic Appearance in Child harboring WT1 variant

Figure/Table	Title	Page
Supplementary Figure S1	Clinical course of the patient (II-3) during the first 7-day of admission	2
Supplementary Figure S2	Imaging studies of the affected child (II-3) on admission	3
Supplementary Figure S3	Profiling of the Copy number variation throughout the genome in affected child (II-3)	4
Supplementary Figure S4	Structural modeling for DNA binding of WT1 variants	5
Supplementary Figure S5	Localization of WT1 Mutations Affecting the Zinc Finger Domain (Exons 7–10)	6
Supplementary Figure S6	Comparison of the cystic kidney gene coverage between the G4500 Panel and Cystic Disease Panel	7
Supplementary Figure S7	Hypothetical pathogenic mechanisms of cystic kidney appearance in our case with p.Arg467Gln	8

Supplementary Table S1	Laboratory findings of the proband (II-3)	9
Supplementary Table S2	Interpretation of identified variants according to ACMG criteria	10
Supplementary Table S3	Comparison of ultrasound findings among the cystic kidney diseases	11
Supplementary Table S4	Characteristics of patients with the p.Arg467Gln variant reported to date	12

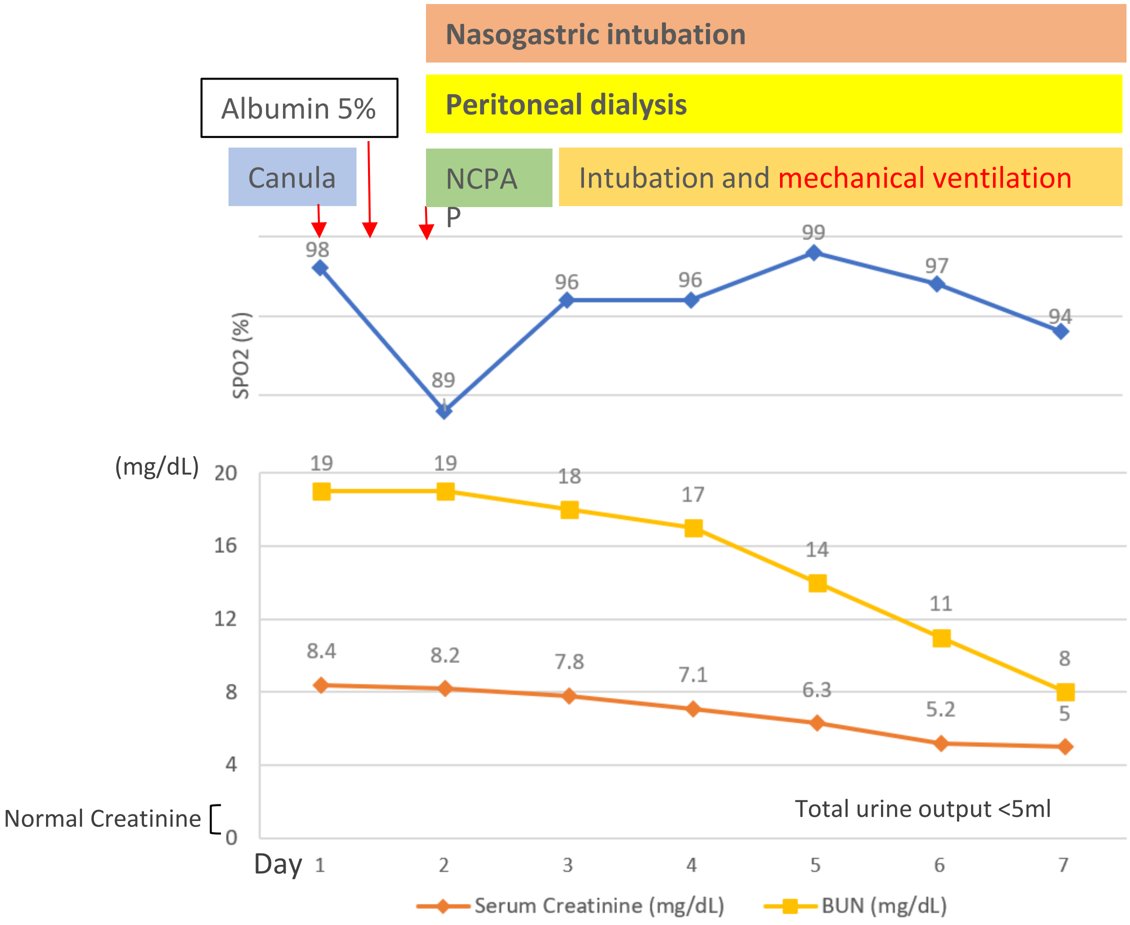

Supplementary Figure S1. Clinical course of the patient (II-3) during the first 7-day of admission.

The chart depicts changes in profiles of serum creatinine, blood urea nitrogen (BUN), and oxygen saturation (SpO_2) during the first 7 days. Renal parameters improved with peritoneal dialysis and albumin therapy. The SpO_2 fluctuations reflect initial respiratory compromise, transiently improved with Nasal Continuous Positive Airway Pressure (NCPAP) but stabilized only after mechanical ventilation.

A

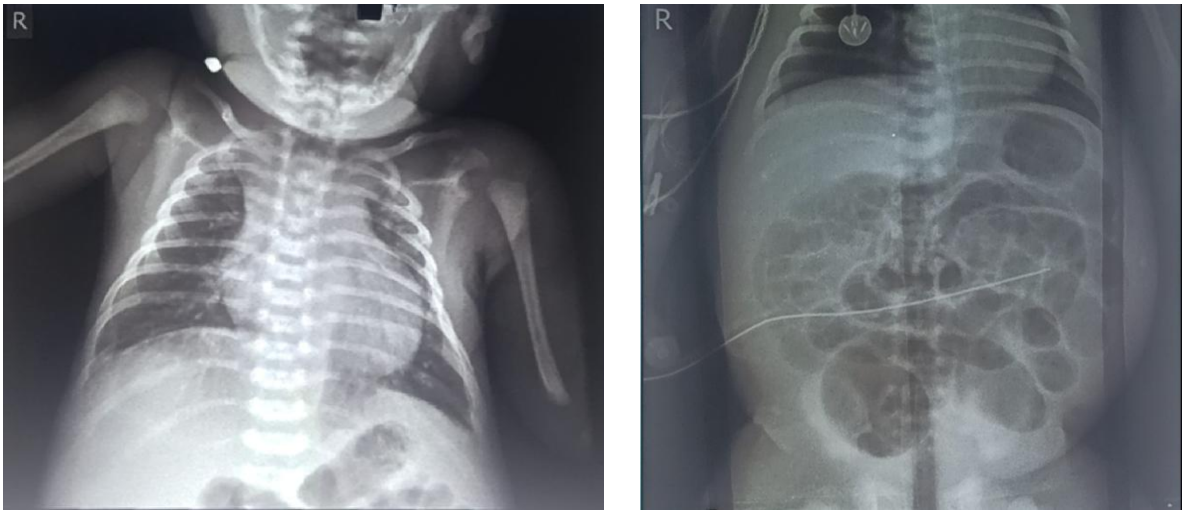

B

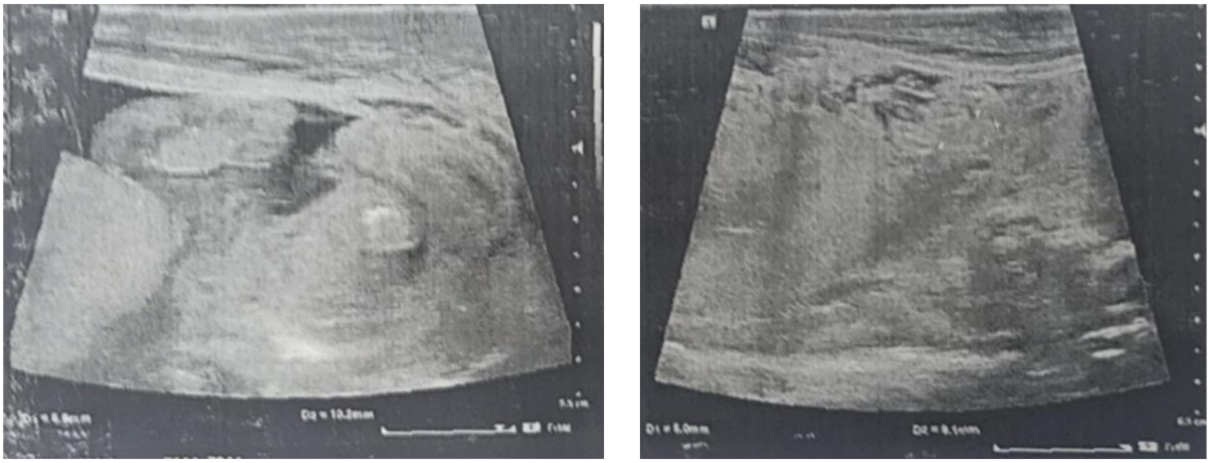

Supplementary Figure S2. Imaging studies of the affected child (II-3) on admission.
A. Chest and abdomen X-ray at age 22-day. There were mild pleural effusion without skeletal abnormalities. Mild gaseous dilatation of the gastrointestinal tract was observed. **B. Ultrasonographic findings of the abdomen at age 22-day**. Transverse scan revealed the undescended testes in the abdominal cavity, right testicle 6x10 mm in diameter, left testicle 5x9 mm without any sign of blood perfusion in tunica vaginalis. Ascites were moderately accumulated in the abdominal cavity, suggesting the fluid retention in third space due to renal dysfunction.

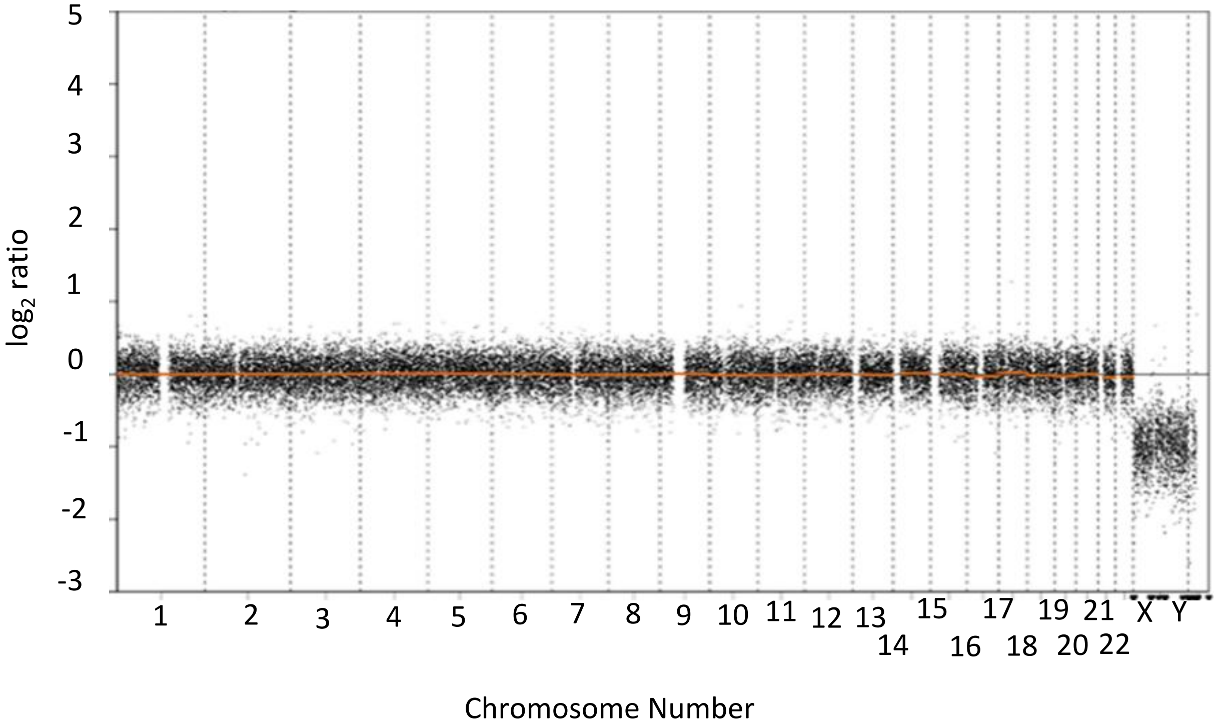

Supplementary Figure S3. Profiling of the Copy number variation throughout the genome in affected child (II-3).

The CNV plot of the affected child (II-3) is shown, with the y-axis representing log_2-transformed normalized sequencing read densities and the x-axis showing sequential 5-kb bins across 60-kb windows. CNV-seq scatter analysis revealed no copy number variants, including at 11p13, the locus of the *WT1* gene.

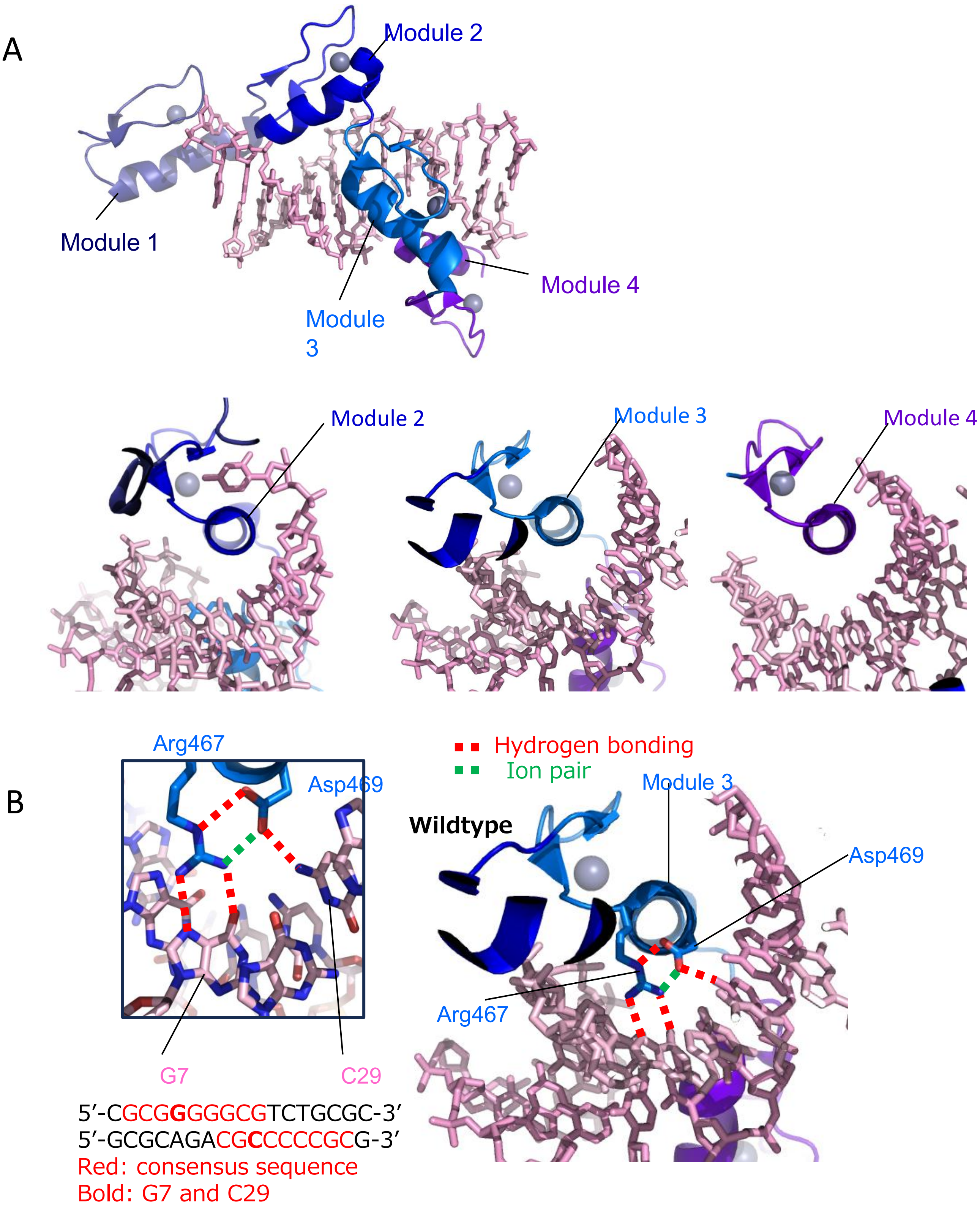

Supplementary Figure S4. Structural modeling for DNA binding of WT1 variants.
A, Overview. AlphaFold2 predicts the positions of Arg467 in relative to the third Zinc finger 394-522 (PDB:2PRT). Interaction of DNA with the Zinc finger motif: The α helices of Module (Zinc finger) 2,3, and 4 intercalate with the major groove of the target DNA. The side chains of amino acid residues in the α helices interact the target DNA sequences. **B, Binding interface of Arg467 and Asp469.** The Arg467 locates in the N-terminal of the Module 3 and interact with the DNA strand through two hydrogen bonds. The neighboring Asp469 interacts with the DNA strand as well as Arg467 side chain through the hydrogen bond and ion-pair. The 467Arg to Gln substitution could interfere with the DNA interaction, by both direct binding between Arg467-DNA and indirect inter-residue interaction (Asp467-Asp469).

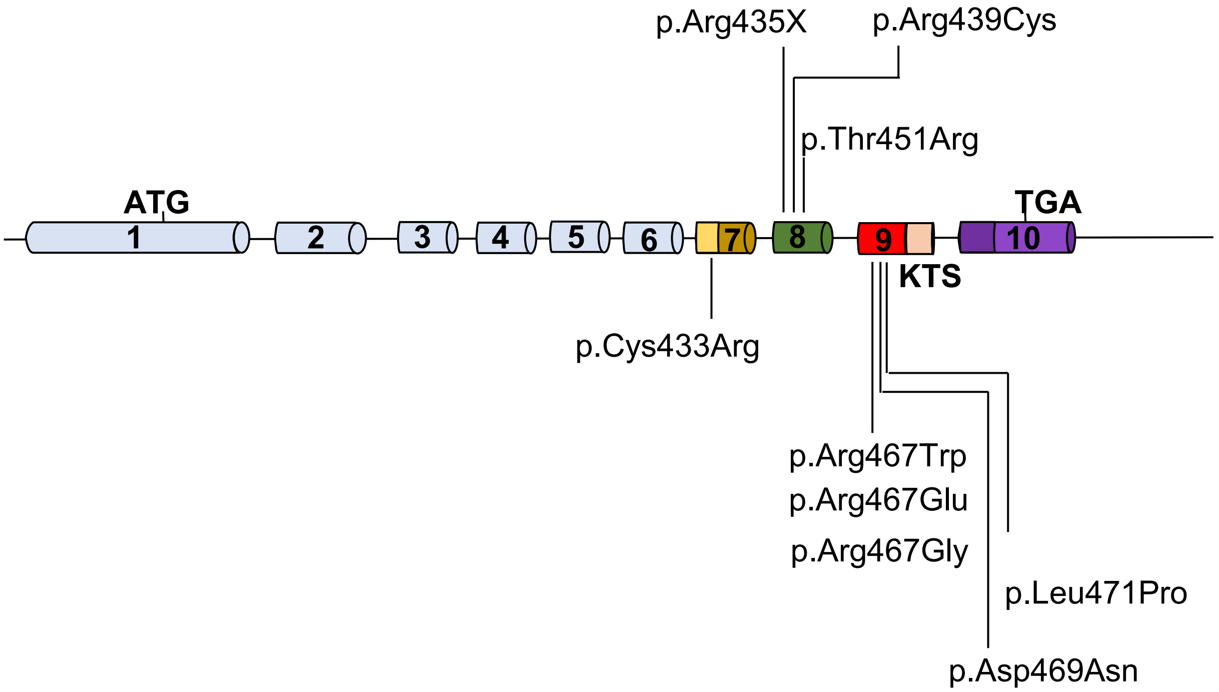

Supplementary Figure S5. Localization of WT1 Mutations Affecting the Zinc Finger Domain (Exons 7–10).
Schematic representation of the *Wilms tumor 1* (*WT1*) gene illustrating the positions of reported pathogenic variants located within the zinc finger region encoded by exons 7–10. Missense and nonsense mutations identified in the literature are mapped to their corresponding positions within exons 7–10, including p.Cys433Arg, p.Arg435X, p.Arg439Cys, p.Thr451Arg, p.Arg467Trp, p.Arg467Glu, p.Arg467Gly, p.Asp469Asn, and p.Leu471Pro (2,6,13-15,20). The reference sequence used for amino-acid numbering is the NCBI 522-aa WT1 isoform D (NM_024426.6; NP_077744.4). This figure highlights the clustering of disease-associated mutations within the C2H2 zinc finger motifs crucial for WT1 DNA-binding function.

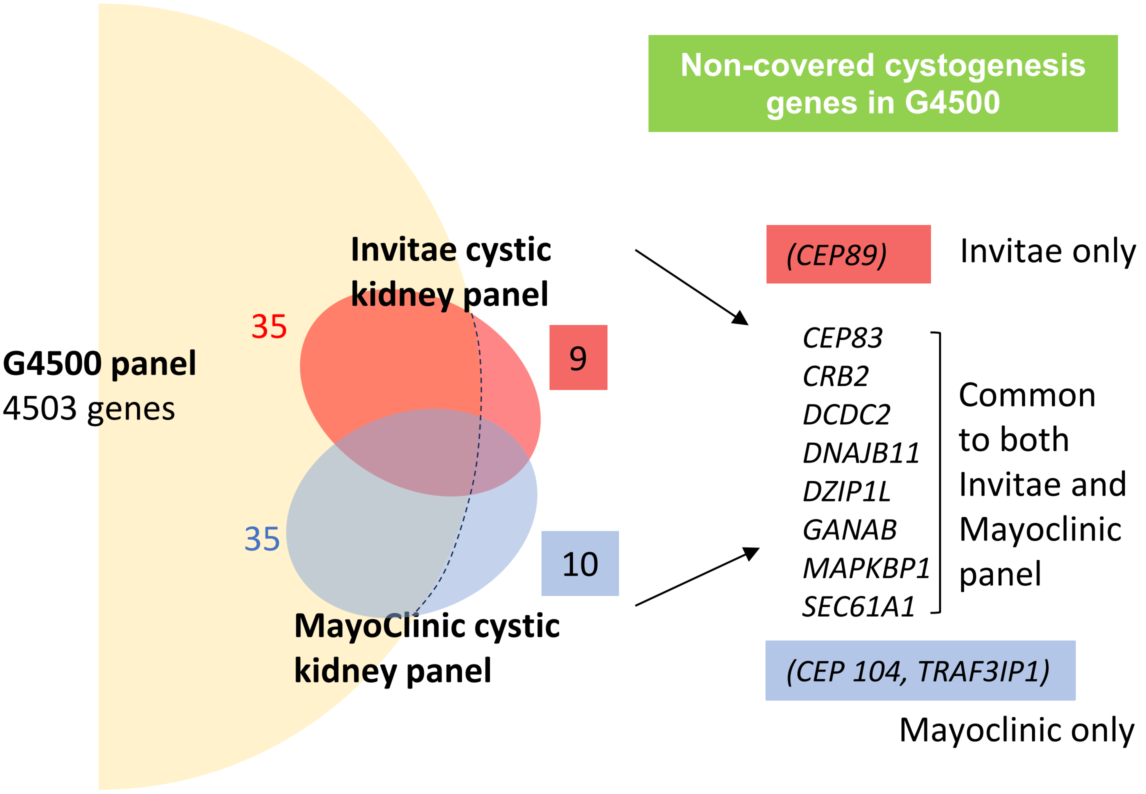

Invitae cystic kidney panel (total n=44)

ALG8	*DNAJB11*	*NEK8*	*SDCCAG8*
ANKS6	*DZIP1L*	*NOTCH2*	*SEC61A1*
BICC1	*GANAB*	*NPHP1*	*SEC63*
CEP164	*GLIS2*	*NPHP3*	*TMEM67*
CEP290	*HNF1B*	*NPHP4*	*TSC1*
CEP83	*IFT172*	*OFD1*	*TSC2*
CEP89	*INVS*	*PAX2*	*TTC21B*
COL4A1	*IQCB1*	*PKD2*	*UMOD*
CRB2	*JAG1*	*PKHD1*	*VHL*
DCDC2	*LRP5*	*PRKCSH*	*WDR19*
DICER1	*MAPKBP1*	*RPGRIP1L*	*ZNF423*

MayoClinic cystic kidney panel (total n=45)

ALG8	*DNAJB11*	*NOTCH2*	*SDCCAG8*
ALG9	*DZIP1L*	*NPHP1*	*SEC61A1*
ANKS6	*GANAB*	*NPHP3*	*SEC63*
BICC1	*GLIS2*	*NPHP4*	*TMEM67*
CEP83	*HNF1B*	*OFD1*	*TRAF3IP1*
CEP104	*INVS*	*PAX2*	*TSC1*
CEP164	*IQCB1*	*PAX2*	*TSC2*
CEP290	*JAG1*	*PKD1*	*TTC21B*
COL4A1	*LRP5*	*PKD2*	*UMOD*
CRB2	*MAPKBP1*	*PKHD1*	*WDR19*
DCDC2	*NEK8*	*RPGRIP1L*	*WDR35*
			XPNPEP3

Invitae only   MayoClinic only

Supplementary Figure S6. Comparison of the cystic kidney gene coverage between the G4500 Panel and Cystic Disease Panel.

The diagram illustrates the cystic kidney genes, which might have been missed by our G4500 Panel analysis: G4500 panel containing 4,503 genes, which covers a majority of previously known inherited cystic disorders disorders (n=50) such as *PKD1, PKD2, PKHD1, ALG8, ALG9, TTC21B, NEK8,* and *INVS.* For these 50 genes, we did not find any mutations. The gene lists of Invitae and MayoClinic panel for cystic kidney disease are shown for comparison. A total of 35 genes are shared by the G4500-Invitae panels as well as G4500-MayoClinic. On the other hand, while 9 genes (Invitae) and 10 genes (MayoClinic) have not been included in our G4500 panel. Therefore, whole exome sequence analysis may allow evaluation of these untested genes and identify unknown cystogenic modifiers in our case.

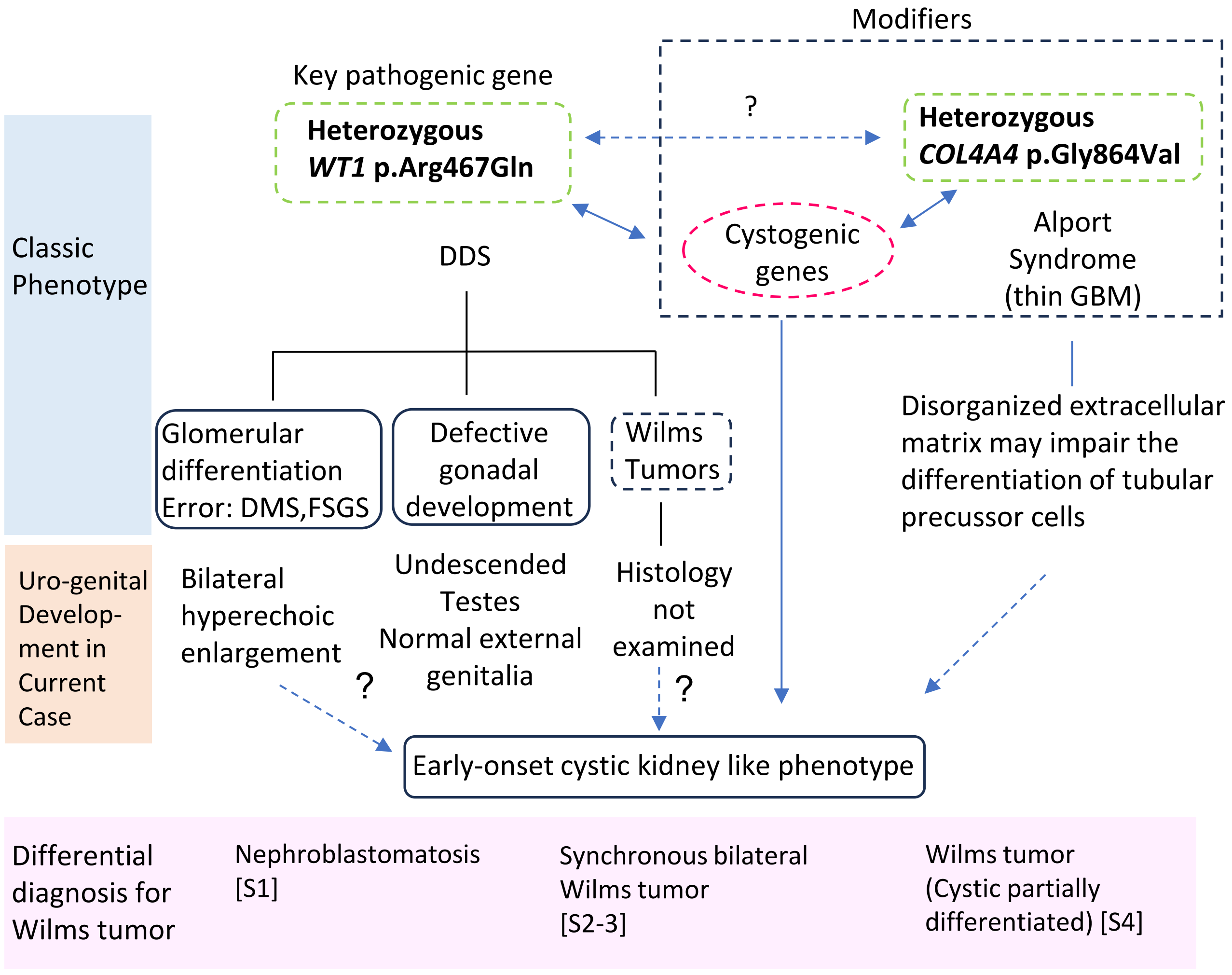

Supplementary Figure S7. Hypothetical pathogenic mechanisms of cystic kidney appearance in our case with p.Arg467Gln.

Our case with *WT1* and *COL4A4* exhibited a unique clinical phenotype of gonadal defects with early-onset cystic kidney. The observation suggests that *WT1* variant may act as a potential modifier of inducing cystic kidney phenotypes through interaction between *WT1* and *COL4A4* or other hidden cystic genes**.**

The oncogenic status of our case could not have been investigated, because of the earlier death. Nephroblastomatosis, which consists of immature, metanephric tissue (nephrogenic rests) is a precursors of Wilms tumors.

DDS, Denys-Drash syndrome; ADPKD, autosomal dominant polycystic kidneys; DMS, Diffuse Mesangial Sclerosis; FSGS, Focal Segmental Glomerulosclerosis.

Supplementary Table S1. Laboratory findings of the proband (II-3).

Variable	On admission<br>At 23 day-old	Reference Range*<br>0-30 days
**Complete Blood Count**		
Hemoglobin (g/dl)	**9.4**	15.0-24.0
Hematocrit (%)	**26.4**	44-70
Mean Corpuscular Volume (fl)	**84.1**	99-115
Mean Corpuscular Hemoglobin (pg)	**29.9**	33-39
White blood cell ($10^3/\mu L$)	13.6	9.1-34.0
Platelet ($10^9/\mu L$)	413	150-400
**Electrolytes & Biochemistry**		
Albumin (g/dL)	1.9	1.9-4.9
Protein (g/dL)	**2.9**	4.6-7.4
Sodium (mmol/L)	**105.8**	134-144
Potassium (mmol/L)	**6.4**	3.4-6.0
Chloride (mmol/L)	**87**	98-106
Phosphorus (mmol/L)	**3.3**	1.25-2.10
Calcium (mmol/L)	**0.8**	1.12-1.23
BUN (mmol/L)	**8.9**	0.84-2.99
Creatinine (μmol/L)	**734**	8.84-31.82**
**Arterial Blood Gas**		
pH	**7.2**	7.35-7.45
pO_2 (mmHg)	98	70-100
pCO_2(mmHg)	**26**	27-41
HCO_3 (mEq/L)	**8.9**	21-28
BE (mEq/L)	-19.8	

Complete blood count, electrolytes, biochemistry, and blood gas analysis were done on admission of postnatal 22 days. The patient suffered from a microcytic hypochromic anemia, and hypoproteinemia. Abnormal electrolytes might be due to severe continuing vomiting and diarrhea before admission, hypoxia and renal dysfunction.
BUN (mmol/L) = Urea (mmol/L) / 2.1428
**Reference range from Nelson Textbook of Pediatrics, 21th Edition*
***Reference range from The Harriet Lane Handbook, 22nd Edition*

Supplementary Table S2. Interpretation of identified variants according to ACMG criteria [S5]

Amino acid change (Zygosity)	Minor Allele Frequency [a)]	Combining criteria for pathogenicity	ACMG codes	ACMG classification
WT1 p.Arg467Gln (Heterozygous)	G=0.00000 (n=8318)	1 Very strong, 2 Strong and 1 Supporting	PP5; PM1, PM2, PM5	Pathogenic
COL4A4 p.Gly864Val (Heterozygous)	T=0.000 (n=112)	1 Strong, 1 Moderate and 2 Supporting	PP3; PM1, PM2, PM5	Likely pathogenic
CEP290 p.Asp2396Tyr rs189556433[b)] (Heterozygous)	Global A=0.0058, (n=140,200), East Asian A=0.0173 (n=3,124)	1 Supporting	PM2	Likely Benign
NPHP1 p.Glu621Gln rs780427871 [c)] (Heterozygous)	Global G=0.000043, (n=140,200), East Asian G=0.0019 (n=3,124)	1 Supporting	PM2	Likely Benign/VUS

PM, pathogenic moderate; PP, pathogenic supporting
PP3: multiple lines of computational evidence support a deleterious effect on the gene or gene product (conservation, evolutionary, splicing impact, *etc*)
PM1: Located in a mutational hot spot and/or critical and well-established functional domain (e.g. active site of an enzyme) without benign variations
PM2: Absent from controls (or at extremely low frequency if recessive) in large population databases.
PM5: Novel missense change at an amino acid residue where a different missense change determined to be pathogenic has been seen before.
[a)] Minor Allele Frequency were refereed to those for the Global and Asian population in gnomAD database
[b)] [c)] Variants have not yet been registered in HGMD

Supplementary Table S3. Comparison of ultrasound findings among the cystic kidney diseases.

Disease feature	ARPKD	Early onset ADPKD (prenatal)	Nephro-blastomatosis	Collagen type IV Disorders
Gene	*PKHD1*	*PKD1/2*	*WT1*	*COL4A3,4,5* (*COL4A1*[#])
OMIM	606702	601313 (PKD1)<br>173910 (PKD2)	607102	120070 (*COL4A3*)<br>120131 *(COL4A4)*<br>303630(*COL4A5*)<br>120130 (*COL4A1*)
Age onset	Prenatal to neonate	Prenatal to childhood	Early childhood	Adult
Origin	Distal tubules and Collecting ducts	All segments of nephron, mainly distal to collecting ducts	Nephrogenic rests	Not determined
Cyst morphology	Numerous micro cysts	Variable size cysts, macroscopic cysts.	No cysts or only few	Oligo to Polycystic
Kidney Enlargement (over average)	Markedly enlarged (+ 4-6 SD)	Moderately (+1-2 SD)	Slightly enlarged	Slightly enlarged or normal size
Echogenecity	Hyper-echogenecity (Homogenous)	Generally hyperechogenic cortex and hypoechogenic medulla	Nephroblastomato sis is shown as hypoechogenic or iso-echogenic foci	Remaining parenchyma is normal
Cortico-medullary differentiatio n (CMD)	CMD Lost	Mostly increase (~50%), may decrease when RF is severe	May vary depend on the severity	May vary depend on the severity
Amniotic fluid	Oligo-hydramnios	Normal volume	Normal	Normal
References	[S6] Burgmaier *Gene Reviews* 2024<br>[S7] Euser *J* Perinatology 2015<br>[S8] Bergmann *Pediatr Nephrol* 2014	[S9] Brun *Ultrasound Obstet Gynecol* 2004<br>[S7] Euser *J Perinatology* 2015<br>[S10] Muto *Nat Com* 2022	[S1] Rohrschneider *Pediatr Radiol* 1998<br>[S12] Dumba *Cancer Imaging* 2015<br>[S13] Wagner *Pediatr Nephrol* 2008	[29] Gulati *Kid Int Rep* 2020<br>[S14] Sevillano *Clin Kidney J* 2014

ARPKD and ADPKD are common forms of the polycystic kidney disease. ARPKD is typically present in infantile/pediatric period, meanwhile ADPKD manifest perinatally on rare occasion. Approximate 1-2% of total ADPKD show phenotype indistinguishable from ARPKD. ADPKD, Autosomal dominant polycystic kidney disease; ARPKD autosomal recessive polycystic kidney disease. RF, Renal failure. CMD, Corticomedullary differentiation. SD, Standard deviation.
