# Supplementary material for: Case Report: Neonatal nephropathy with polycystic appearance in child harboring WT1 variant
Source: Front Pediatr. 2025 Dec 10;13:1693036. doi: 10.3389/fped.2025.1693036 (PMC12728025; doi:10.3389/fped.2025.1693036)
Supplement: Supplementary file 2 [file Presentation2.pdf]

Supplementary Table S4. Characteristics of patients with the p.Arg467Gln variant reported to date

Author (year)	Our case	Yoshino (2022)	Maalouf (1998)	Nagano (2021)	Kohler (2011)	Chernin (2010)		Jeanpierre (1998)	Schumacher (1998)
Publication		Ref [20]	Ref [21]	Sup Ref [S15]	Sup Ref [S16]	Ref [6]		Ref [2]	Sup Ref [S17]
Patient ID				Neph248	5	A1276	A2072	P15	NS5
Karyotype	46XY	46XY	46XY	46XX	46XY	46XX	46XY	46XY	46XY
Onset proteinuria (year)	0	0	0	0.3	At birth	0	0	0.4	0.01
Age of ESRD (year)	0	0	0	0.3	At birth	0.1	0.1	0.6	0.33
Age of death (age, day)	47		15 days						
Renal Histology	*nd*	*nd*	*nd*	*nd*	*nd*	CNS	*nd*	*DMS*	FSGS
Onset of Wilms Tumors (year)								U, 0.7	nd
Birth weight (g)	2500	2558	3400	*nd*	*nd*	*nd*		*nd*	*nd*
**US**									
Kidney size (cm, long, short axes)	Enlarged R 5.4x3.2 L 5.3x3.6 (2-3SD)	Smaller R 3.0x2.0 (-3 to -4SD) L 3.8x1.8 (-1SD)	enlarged	*nd*	*nd*	*nd*		*nd*	*nd*
Cysts	PKD-like	No cyst	No cyst	*nd*	*nd*	*nd*		*nd*	*nd*
Echogenicity	Increased CMD↓	Increased	Increased	*nd*	*nd*	*nd*		*nd*	*nd*
**MRI**			CMD↓						
Examined age	*nd*	Fetus 33W	Day 3	*nd*	*nd*	*nd*		*nd*	*nd*
Images		No cysts	Enlarged dysplastic, poor CMD						
**Other clinical features**									
	Cryptorchidism PKD-like appearance Sib affected	Potter syndrome, Ambiguous external genitalia	Potter syndrome		Penoscrotal hypospadias, hypoplastic phallus, hypoplastic scortum, bilateral cryptorchidism			Testicular ectopia, no nephrectomy	Hypospadias, cryptorchidism micropenis

Clinical phenotypes previously described for the patients with WT1 p.Arg467Gln variant are summarized.
ESRD, End stage Renal Disease; CMD, Cortico-medullary differentiation. CNF, congenital nephrotic syndrome of the Finnish type; CKD 5, chronic kidney disease stage 5 (eGFR <15 ml/min/1.73m^2)
DMS, diffuse mesangial sclerosis; FSGS, focal segmental glomerulosclerosis; U, unilateral
IF, interstitial fibrosis; nd, not determined
